# Arterio-venous gradients of IL-6, plasma and serum VEGF and D-dimers in human cancer

**DOI:** 10.1038/sj.bjc.6600655

**Published:** 2002-12-02

**Authors:** R Salgado, I Benoy, R Weytjens, D Van Bockstaele, E Van Marck, Ph Huget, M Hoylaerts, P Vermeulen, L Y Dirix

**Affiliations:** Angiogenesis Group, Oncology Center, St.-Augustinus Hospital, Oosterveldlaan 24, 2610 Wilrijk, Belgium; Department of Pathology, University of Antwerp and University Hospital of Antwerp, Universiteitsplein 1, 2610 Wilrijk, Belgium; Laboratory of Hematology, University of Antwerp and University Hospital of Antwerp, Universiteitsplein 1, 2610 Wilrijk, Belgium; Center of Molecular and Vascular Biology, University of Leuven, Herestraat 49, 3000 Leuven, Belgium

**Keywords:** D-dimers, fibrinolysis, VEGF-A, interleukin-6

## Abstract

The circulating angiogenic factors vascular endothelial growth factor-A, interleukin-6 and the fibrin D-dimer fragment were measured in the mesenteric vein, the uterine vein, as well as in peripheral venous and arterial samples in 21 randomly selected patients with operable colorectal, ovarian and cervical carcinoma. In addition, immunohistochemistry for vascular endothelial growth factor-A and interleukin-6 was performed on colorectal tumours of such patients. Serum and plasma vascular endothelial growth factor-A were not significantly elevated in the vein draining the tumours, despite tumour cell expression of vascular endothelial growth factor-A. Serum vascular endothelial growth factor-A is therefore not all tumour-derived. In contrast, serum interleukin-6 was highly elevated in the draining veins in agreement with expression of interleukin-6 in the cytoplasm of tumour cells. In the megakaryoblastic cell line MEG-01, the expression of vascular endothelial growth factor-A was found to be regulated by interleukin-6. Thus, the higher platelet vascular endothelial growth factor-A load resulting in higher serum vascular endothelial growth factor levels in cancer patients may partly result from an interleukin-6 mediated up-regulation of the expression of vascular endothelial growth factor-A in the precursor of the platelet, i.e. the megakaryocyte. We also confirmed by immunohistochemistry that platelets adhere and aggregate on tumour endothelium. We propose that interleukin-6 indirectly promotes tumour angiogenesis through its up-regulation of the vascular endothelial growth factor-A load in platelets. In addition, the correlations found between peripheral venous interleukin-6 and peripheral venous fibrinogen and D-dimers levels, and the high D-dimer levels found in the draining vein of the tumour, in agreement with fibrin deposits found in the tumour stroma, suggest an important role for interleukin-6 in extra-vascular fibrinogen metabolism. Our results suggest a pivotal role for interleukin-6 in the intrinsic link between haemostasis and angiogenesis. This might be of importance in the development of anti-angiogenic agents based on interference with haemostasis.

*British Journal of Cancer* (2002) **87**, 1437–1444. doi:10.1038/sj.bjc.6600655 www.bjcancer.com

© 2002 Cancer Research UK

## 

Haemostasis and angiogenesis are tightly regulated physiological processes involved e.g. in wound healing and deregulated in cancer development (Carmeliet and Jain, 2000). Both may be critical in three inter-related features of tumour progression, i.e. growth, invasion and metastasis (Carmeliet and Collen, 1998).

Vascular Endothelial Growth Factor-A (VEGF-A) is one of the most important positive mediators of angiogenesis. Tumour cells as well as stromal cells express VEGF-A (Leung *et al*, 1989; Veikkola *et al*, 2000). VEGF-A not only regulates endothelial cell migration, proliferation and survival, but is also known as Vascular Permeability Factor (VPF) because it enhances endothelial cell permeability even more potently than histamine (Dvorak *et al*, 1995). VEGF-A also induces tissue factor on endothelium and tumour cells, activating coagulation and fibrin formation (Mechtcheriakova *et al*, 1999). This is of primary importance for angiogenesis and tumour growth (Nagy *et al*, 1989; 1995). High serum levels of VEGF-A are predictive for tumour doubling time in patients with colorectal cancer. Elevated levels nearly normalise after resection of the primary tumour (Dirix *et al*, 1997). These observations indicate that circulating levels of VEGF-A mirror the ongoing growth process in tumours.

A nearly 10-fold higher serum than plasma VEGF-A level is found in both cancer patients as in healthy persons (Banks *et al*, 1998), indicating that circulating VEGF-A is mainly blood cell associated, in particular in platelets (Verheul *et al*, 1997; Salgado *et al*, 1999; Gunsilius *et al*, 2000). High serum VEGF-A levels are frequently encountered in cancer patients, the platelets of whom indeed have a higher VEGF-A content compared with healthy individuals; a prognostic significance is attributed to these levels (Salven *et al*, 1999; O'Byrne *et al*, 2000). How VEGF-A is regulated in platelets or in megakaryocytes in cancer patients is unclear, but VEGF-A in epithelial cells is regulated by IL-6 (Cohen *et al*, 1996). Platelet production is also regulated by IL-6 (Ishibashi *et al*, 1989).

The interstitial fibrin matrix, formed by VEGF-A-mediated tissue factor expression on tumour and endothelial cells, or by shedding of cancer pro-coagulant proteins (Mielicki *et al*, 1990), may further serve as a scaffold for endothelial cell migration and proliferation (Contrino *et al*, 1997). In addition, fibrin-(ogen) degradation products have pro-angiogenic properties (Thompson *et al*, 1985; Stirk *et al*, 2000). Remarkably, high fibrinolytic derivatives are one of the most frequent coagulation test abnormalities encountered in the oncological setting (Edwards *et al*, 1987).

In the present work we measured the circulating factors VEGF and IL-6 in veins draining the tumour in order to provide direct evidence of tumour secretion of these factors. We further investigated how platelets of cancer patients acquire a higher VEGF-A content. We then analysed whether circulating fibrin degradation products, D-dimers, are derived from intra-tumoural activation of haemostasis. We more specifically focused on the role IL-6 could have in both increasing the VEGF-content in platelets as well as in mediating fibrin formation in tumours.

## MATERIALS AND METHODS

### Patients

Two patients with cervical carcinoma (FIGO Ib), five with ovarian carcinoma (FIGO III), two of them with extensive disease (FIGO IIIc), and 14 with colorectal carcinoma (Dukes A–C), of which four had extensive disease at the moment of surgery (Dukes D), were scheduled for elective abdominal surgery. Exclusion criteria were: previous, concurrent or non-resectable cancer, local or systemic inflammatory disease. The following staging procedures were performed: chest X-ray, bone scintigraphy, CT-scan and echography of the liver. All patients received 0.3 mg fraxiparin subcutaneously for prevention of deep venous thrombosis (DVT). All routine precautions for prevention of DVT were taken.

### Tissue

As part of the normal surgical resection procedure, of the main tumour mass, a representative, full cross section of the tumour sample surrounded by adjacent mucosa was taken from all patients involved. The tissue was fixed in buffered formalin and paraffin-embedded. Five μm sections were cut and mounted on poly-L-lysine coated slides. Successive sections were used for immunostaining.

### Blood coagulation tests

During the surgical procedure, blood sampling occurred simultaneously in the mesenteric or uterine vein as well as in the brachial vein and brachial artery respectively. Plasma collection and measurement of prothrombin time (PT), activated partial thromboplastin time (aPTT), fibrinogen, platelet counts and D-dimer levels were performed as mentioned previously (Dirix *et al*, 2002).

## Elisa

### 

#### Angiogenic cytokines

Blood sampling was performed as mentioned above. Serum collection and VEGF-A_165_ and IL-6 measurements were performed as mentioned previously (Salgado *et al*, 1999). Plasma VEGF-A_165_ was measured with an ELISA-kit of R&D systems (R&D systems, Minneapolis, MN, USA) on trisodium citrate-anticoagulated collected blood. For the ELISA-assay of VEGF-A_165_, no cross-reactivity with other members of the VEGF-A family is documented. Samples were assayed in duplicate. Within assay variability has been tested before (Salgado *et al*, 1999).

### Immunohistochemistry

#### VEGF

Immunohistochemistry was performed on colorectal cancer tissue as described previously with the anti-VEGF-A VG-1 antibody that stains VEGF-A efficiently on paraffin sections (Turley *et al*, 1998). This antibody recognises the 121, 165 and 189 isoforms of the VEGF-A protein.

#### IL-6

Deparaffinisation and quenching of endogenous peroxidase was performed. The primary 1/10 diluted polyclonal anti-IL-6 antibody (Genzyme Co., Cambridge, MA, USA) was then applied on sections of the colorectal carcinomas followed by a biotinylated secondary antibody. Streptavidin conjugated with peroxidase was subsequently applied on the sections (DAKO, Glostrup, Denmark). Finally, amino-ethylcarbamazimine (AEC) was used as a substrate reagent. Negative controls included the omission of the primary antibody and the use of an irrelevant primary antibody.

#### Fibrin

After deparaffinisation and quenching of endogenous peroxidase, protein digestion was performed with protease. Slides were placed on a Ventana NexES (Ventana Medical Systems, Inc.) automated immunostainer and stained with a 1/100 diluted monoclonal T_2_ G_1_ antibody (Acc. Chem. Sci. Corp., USA) overnight at 4°C. This antibody is specific for the peptide Bα 15-42 of fibrin and for fibrin II. Fibrin II is obtained after cleavage of fibrinogen by thrombin at the bond Aα 16-17 and Bβ 14-15. Fibrinopeptides A and B are released after cleavage by thrombin. Consequently, intact fibrinogen was not stained (Kudryk and Bini, 2000). The staining was completed with a Vector Basic DAB detection kit (Ventana Medical Systems, Inc.). Negative controls included the omission of the primary antibody. Paraffin embedded placental tissue with peri-villous fibrin deposits was used as a positive control.

#### Platelets

Immunohistochemistry for platelet glycocalicin was performed to demonstrate adherence and aggregation of platelets on endothelium in colorectal tumours. Glycocalicin is a fragment of the platelet membrane glycoprotein Ib (Steinberg *et al*, 1987).

Deparaffinisation and quenching of endogenous peroxidase was performed. Normal rabbit serum (NRS), diluted 1/5, was applied, followed by 1/20 000 diluted primary mouse anti-human glycocalicin (clone: G28E5) antibody (kind gift from Dr Hoylaerts). Subsequently, the 1/400 diluted, secondary biotinylated rabbit anti-mouse antibody (DAKO, Glostrup, Denmark) was applied. The immune reaction was completed with ABC-streptavidin detection system and DAB (diaminobenzidine) as chromogen. The slides were counterstained with haematoxylin and mounted. Negative controls included the omission of the primary antibody, the addition of an irrelevant primary antibody directed against glucose-oxidase from *Aspergillus* Niger species (this enzyme is not inducible in mammalian cells) and paraffin embedded normal endometrium tissue. Positive controls included staining of platelets in paraffin embedded thrombus and placenta with prominent fibrin deposits as well as in a platelet pellet.

### Cell culture experiments

We used the human megakaryoblastic cell line MEG-01 to evaluate whether IL-6 is able to modulate the expression of VEGF in megakarycocytes. Ogura and colleagues established the MEG-01 cell line from a patient in a megakaryoblastic crisis of chronic myelogenous leukaemia. This cell line possesses many megakaryocytic specific markers and does not posses any marker of B-cells, T-cells and myeloid cells (Ogura *et al*, 1985).

The MEG-01 cell line has the receptor for IL-6 and blocking IL-6 with anti-IL-6 antibodies did not have any significant effects on cell growth (Kellar *et al*, 1995). In order to confirm this we analysed with the Alamar blue assay (Trek Diagnostics, USA) whether alterations in cell proliferation occur after adding IL-6 (10 and 100 ng ml^−1^) to the culture medium. The cells were cultured in Iscove's modified Dulbecco`s medium (IMDM), with 10% foetal calf serum and antibiotics. The experiments were initiated by seeding 5×10^4^ cells well^−1^ in a 24-well plate and allowed to proliferate for 24 h in 10% FCS medium before adding 10 or 100 ng ml^−1^ IL-6 in addition with 1/10 diluted Alamar blue medium. Fluorescence intensities, as a measure of reduction of the Alamar blue medium by proliferating cells, was measured every 6 h with a Cytofluor (Cytofluor™ 2300, Millipore).

In subsequent experiments the IL-6 receptor (IL-6R) was blocked for 48 h with a molar excess of monoclonal anti-IL-6R antibody (20 μg ml^−1^) (R&D Systems, Minneapolis, MN, USA). Cells without antibody served as negative controls.

Cell viability was assessed using Trypan blue exclusion dye. Conditioned medium was collected and ELISA for VEGF-A was performed. Experiments were performed in triplicate.

### Ethical committee

The local Ethical Committee approved this study and informed consent was obtained from all patients involved.

### Statistical analysis

Statistical analysis was performed with Graphpad Prism, version 2.0 (Graphpad Software, Inc.). Half the detection limit value of the patient samples was used for statistical analysis in case the measured values did not reach the detection limit of the assay. Detection limits were 0.7 pg ml^−1^ and 9 pg ml^−1^ for the IL-6 and VEGF-A assays, respectively. Comparisons of continuous variables were performed with the Mann–Whitney *U*-test. A paired students *t*-test was used to evaluate observed differences in the cell culture experiments. The relation between continuous variables was analysed with a Spearman rank correlation analysis. Means±standard deviations are given. A *P*-value <0.05 was considered to be significant.

## RESULTS

### Analysis of VEGF, IL-6 and D-dimers in patients without distant metastasis

In order to verify whether there was a gradient for VEGF, IL-6 and D-dimers between the afferent and efferent blood flow in a tumour we explicitely used samples from patients without distant metastasis (*n*=15) as VEGF, IL-6 and D-dimers produced by metastases could mask observed gradients. We also assumed that the values in the brachial artery samples are comparable with mesenteric and uterine artery samples. Mean age of all patients involved is 65.71±13.4 years.

#### VEGF-A, serum and plasma VEGF-A

**Figure 1:**
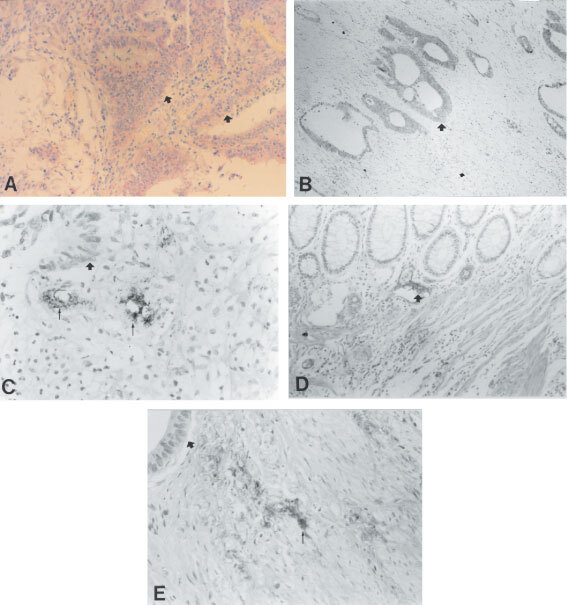
(**A**) VEGF-immunoreactivity in colorectal tumour cells (arrows ×400). (**B**) Interleukin-6-immunoreactivity in colorectal tumour cells (arrow ×400). (**C**) Adherence, aggregation and extravasation of platelets (thin arrows) in the vicinity of colorectal tumour cells (thick arrows ×400). (**D**) Intra-vascular immunostaining of fibrin on endothelial cells (arrow ×400). (**E**) Extra-vascular stromal staining (thin arrows) of fibrin near tumour cells (thick arrow ×400).

**Figure 2:**
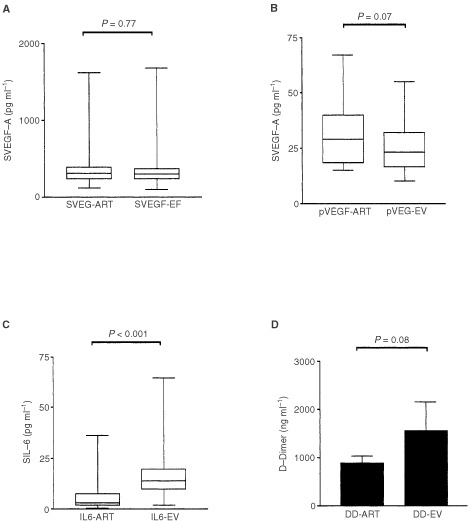
Observed gradients between arterial samples (art.) and tumour efferent blood (EV: efferent vene: mesenteric and uterine vene) for (**A**) serum VEGF-A, (**B**) plasma VEGF-A, (**C**) Interleukin-6 and (**D**) D-dimers in patients with localised cervical, ovarian and colorectal cancer (*n*=15).

Seventy-five per cent of all patients involved with colorectal cancer (*n*=14) had tumour cell expression of VEGF-A (Figure 1A

). We analysed in patients without distant disease (*n*=15) whether tumour cell produced VEGF-A is secreted in the main blood stream, accounting herewith for the high serum VEGF-A levels found in cancer patients. None of the patients had serum or plasma values below the detection limit of the assay. No significant differences were observed between serum VEGF-A in arterial and mesenteric/uterine vein samples (345.2±269.5 pg ml^−1^ and 482.7±371.1 pg ml^−1^ respectively; *P*=0.77; Figure 2A

: *n*=15; Table 1

**Table 1:**
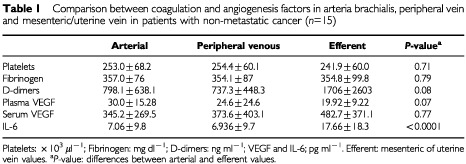
Comparison between coagulation and angiogenesis factors in arteria brachialis, peripheral vein and mesenteric/uterine vein in patients with non-metastatic cancer (*n*=15)

). We compared plasma VEGF between arterial and mesenteric/uterine vein samples. Means of 30±15.28 pg ml^−1^ and 19.92±9.22 pg ml^−1^ were found for arterial and tumoural efferent samples respectively (*P*=0.07; Figure 2B; *n*=15; Table 1).

#### Interleukin-6

All patients with colorectal cancer had extensive IL-6 labelling in the tumour cell compartment (Figure 1B; *n*=14). A 2.5-fold higher serum level of IL-6 was observed in the mesenteric/uterine vein compared with arterial samples (17.66±18.3 *vs* 7.06±9.8 pg ml^−1^; *P*<0.001, Mann–Whitney *U*-test; Figure 2C; *n*=15; Table 1). Only one patient had an IL-6 value below the detection limit of the assay.

**Figure 3:**
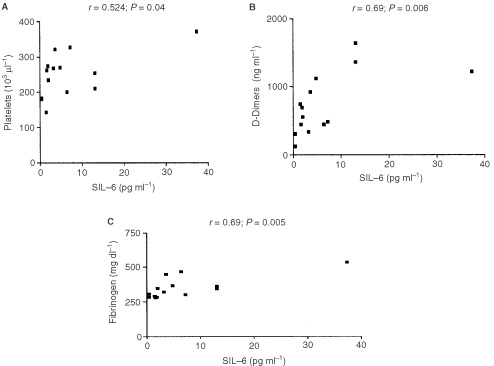
Observed correlation analysis between peripheral venous serum IL-6 and (**A**) platelets, (**B**) D-Dimers and (**C**) fibrinogen (*n*=15).

A correlation was observed between circulating IL-6 (6.9±9.7 pg ml^−1^), and platelet count in the peripheral venous circulation in all patients without metastasis (*r*=0.54; *P*=0.04; Figure 3A

; *n*=15). Also, IL-6 correlates with circulating fibrinogen levels (*r*=0.69; *P*=0.005) and with D-dimers (*r*=0.69; *P*=0.006; Figure 3B,C; *n*=15) in brachial vein samples.

#### Fibrinogen, fibrin and D-dimers (*n*=15)

Fibrinogen levels were 357±76 mg dl^−1^ for the arterial samples, 354.1±87 mg dl^−1^ and 354.8±99.8 mg dl^−1^ for the venous peripheral and tumoural draining vessels respectively (*n*=15). No statistical differences were observed between fibrinogen levels in the arterial and efferent draining vein respectively (*P*=0.79; Table 1).

Mean D-dimer levels in the mesenteric/uterine vein were 1706±2603 ng ml^−1^, whereas in the brachial artery a mean value of 798.1±638.1 ng ml^−1^ was found (*P*=0.08; Mann–Whitney *U*-test; Figure 2D; Table 1; *n*=15). As already mentioned, both aPTT and PT values were in the normal range indicating that the high D-dimer levels encountered in the draining veins are not due to artifactual activation of coagulation during sampling.

The major substrate of D-dimers, fibrin, was encountered both in the stromal as well as on the luminal and/or abluminal side of endothelial cells in the majority of cases examined (Figure 1D,E; *n*=14). No spatial co-localisation between platelet and fibrin staining was evident.

#### Platelets (*n*=15)

Mean platelet count in the brachial vein, brachial artery, and mesenteric/uterine vein were 254.4±60.1× 10^3^ μl^−1^, 253±68.2×10^3^ μl^−1^ and 241.9±60×10^3^ μl^−1^. No statistical differences were observed between platelet counts in the arteria brachialis and efferent draining vein (Table 1; *n*=15). Activated Partial Thromboplastin Time (APTT) en PT were in all samples in the normal range indicating that no artifactual coagulation occurred in the samples taken. To merely verify whether platelet adherence and aggregation occurs in tumours we performed immunohistochemistry using an antibody directed at the platelet membrane protein glycocalicin. Positive staining indicates that these platelets are activated since glycocalicin is translocated to the platelet membrane upon activation.

The specificity of the glycocalicin platelet antibody was demonstrated in the positive controls (platelets in a platelet pellet, in a thrombus and in placental fibrin deposits), where clear staining of platelets was shown. Tumour cells, the fibrin matrix in the thrombus, white blood cells as well as in the placental fibrin deposits stained negative. Platelet adherence and aggregation on endothelial cells and extra-vasation of platelets was observed in all tumours (Figure 1C; *n*=14).

### Analysis of VEGF, IL-6 and D-dimers in patients with distant metastasis

In order to find evidence for IL-6 and D-dimer secretion by metastatic cells we compared arterio-venous differences in the primary tumour of patients with metastasis (*n*=6). If metastatic cells would secrete both IL-6 as well as D-dimers, arterial levels of these would increase and would consequently mask or lessen the arterio-venous gradients as were observed in non-metastatic patients. Mean age of all patients with metastasis involved is 62.8±9.2 years.

### Interleukin-6

Arterial levels of IL-6 were compared in metastatic cancer patients (*n*=6) were compared with arterial levels in non-metastatic patients (*n*=15). Analysing IL-6 in those patients with extensive disease elaborated about two-fold higher arterial IL-6 levels (13.42±17.4 pg ml^−1^) than respectively encountered in those patients without metastatic disease (7.06±9.8 pg ml^−1^). None of the patients had IL-6 values below the detection limit of the assay. Analogous results were obtained when comparing peripheral venous samples (12.95±15.5 pg ml^−1^). When analysing arterio-venous differences in these patients with a mean level of IL-6 of 80.20±129.9 pg ml^−1^ in the mesenteric/uterine vein, a *P*-value of 0.09 compared with *P*<0.0001 in the non-metastatic group was found.

### D-dimers

Arterial levels of D-dimer concentrations of patients with advanced disease (*n*=6) were compared with non-metastatic patients (*n*=15). A three-fold higher arterial D-Dimer level (2372±1741 ng ml^−1^) was found in advanced metastatic patients compared with those patients without metastatic disease (798.1±638.1 ng ml^−1^). Analogous results are obtained when comparing peripheral venous samples (2575±1870 ng ml^−1^). When analysing these arterial levels with the D-dimers measured in the veins draining the primary tumour in these patients (*n*=6; 3066±1741 ng ml^−1^), a *P*-value of 0.69 compared with *P*=0.08 in patients without metastasis was yielded.

### Effect of IL-6 on VEGF-A secretion in the MEG-01 cell line

Cell viability exceeded 98% in all circumstances. The mean IL-6 and VEGF-concentrations (pg ml 1^−1^×10^3^ cells) in the conditioned medium of the control cells were 2.84±2.084 pg ml 1^−1^×10^3^ and 6.093±2.651 pg ml 1^−1^×10^3^ cells, respectively.

Incubating the MEG-01 cells for 48 h with 10 or 100 ng ml^−1^ IL-6 yielded mean VEGF-values of 5.34±1.6 pg ml 1^−1^ and 4.72±0.6 pg ml 1^−1^×10^3^ cells, which were not significantly higher than the VEGF concentration in the conditioned medium of control wells.

We then analysed with the Alamar blue assay whether blocking of the IL-6 receptor resulted in a significant inhibition of cell proliferation. No significant changes in cell proliferation were observed (data not shown).

**Figure 4:**
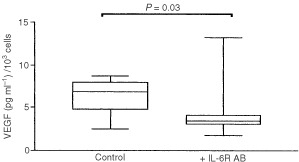
Cell culture experiment in which VEGF-A is measured with ELISA in the conditioned medium of MEG-01 cell lines treated with a blocking antibody against the IL-6 receptor.

The median±s.e. (standard error) VEGF concentration after 48 h incubation with an IL-6R blocking antibody was 3.44±1.44 pg ml 1^−1^×10^3^ compared with 6.850±1.082 pg ml 1^−1^×10^3^ cells for the control cells (*P*=0.03) (Figure 4

).

## DISCUSSION

Interleukin-6 is a cytokine involved in haematopoietic cell differentiation, in the regulation of the acute-phase response, in immune regulation and is produced by epithelial, bone marrow and blood borne cells (Borden and Chin, 1994; Simpson *et al*, 1997; Hirano, 1999). Several studies have elaborated on the prognostic impact of circulating IL-6. A poor prognosis has been consistently associated with high levels of serum IL-6 in patients with renal cell, ovarian, and prostate cancer (Ljungberg *et al*, 1997; Tempfer *et al*, 1997; Nakashima *et al*, 2000). We have shown that high serum levels of IL-6 have independent prognostic significance in a series of 96 patients with metastatic breast cancer (data not shown). de Vita *et al* (2001) have recently demonstrated, in univariate analysis, that high circulating IL-6 levels are associated with reduced overall survival and reduced time to disease progression in patients with gastrointestinal cancer.

Our results demonstrate that circulating IL-6 is mainly derived from the tumour in patients with colorectal, cervical and ovarian cancer (Figure 2C). Moreover, arterial and peripheral venous circulating levels of IL-6 are about two-fold higher in patients with disseminated disease compared with arterial and peripheral venous IL-6 levels of patients with localised disease. These higher levels diminish the arterio-venous differences in the primary tumour of the patients with metastatic disease. This suggests that metastasised cells also produce and secrete IL-6. Nevertheless, to definitively prove that metastases secrete Il-6, gradient studies need to be performed. This study, however, will be difficult to perform due to technical and ethical reasons.

We have also demonstrated in this study that neither serum or plasma VEGF-A levels are significantly elevated in the vein draining the tumour, despite a high tumour cell expression of VEGF-A (Figure 2A,B). This contradicts the common notion that serum VEGF-A is mostly derived from spill over of tumour cell produced VEGF-A in patients with cancer. Landriscina *et al* (1998) also did not find significantly higher serum levels of VEGF-A in mesenteric blood compared with peripheral blood in patients with colorectal cancer. This finding was recently confirmed in patients with rectal cancer (Werther *et al*, 2001). These observations suggest that serum VEGF-A might be derived from other sources. However, it might be possible that continuous spilling of tumour-derived VEGF in the circulation might lead, especially considering the half-life of VEGF-A of about 30 min (Eppler *et al*, 2002), to equivalent levels of venous and arterial VEGF-A.

Several groups have confirmed the role of platelets in the storage of circulating VEGF-A (Verheul *et al*, 1997; Banks *et al*, 1998; Salgado *et al*, 1999; Gunsilius *et al*, 2000; Salgado *et al*, 2001).

In a previous study we have shown that serum IL-6 correlates not only with the platelet count (illustrating its thrombopoietic property), with serum VEGF-A (illustrating the importance of IL-6 in the regulation of VEGF-A), but also with the VEGF-A load in platelets (Salgado *et al*, 1999). Since thrombopoietic cytokines, such as thrombopoietin (TPO) and interleukin-3 (IL-3), enhance VEGF-A expression in megakaryocytes, and, as is the case for TPO, in parallel with megakaryocytic differentiation, we hypothesised that IL-6 derived from primary tumour cells as well as from disseminated tumour cells might enhance VEGF-A expression in platelet progenitors much in the same manner (Imai *et al*, 1991; Mohle *et al*, 1997; Bobik *et al*, 1998; Salgado *et al*, 1999).

Our *in vitro* data demonstrate that the endogenous production of IL-6 in a megakaryocytic cell line, MEG-01, is substantially high. The observation that adding IL-6 did not have any effect on VEGF-A production, might suggest that the IL-6 receptors are already maximally stimulated and saturated. We therefore opted for blocking this autocrine pathway with a molar excess of IL-6 receptor blocking antibody. Our results indicate that VEGF-A production is, at least partly, regulated by IL-6 (Figure 4).

We elaborate on an important role of tumour cell produced IL-6 in the enhanced platelet VEGF-A load of cancer patients explaining herewith the previously found correlation between serum IL-6, VEGF-A and the VEGF-A load in platelets. Moreover, as we did not find higher serum or plasma VEGF-A levels in the efferent blood vessel of tumours, the role of bone marrow, thus megakaryocyte-derived platelet VEGF-A, may contribute more to the total serum VEGF-A than previously suspected. Salven *et al* (1999) have shown that circulating leucocytes contribute only for 17% of the total circulating VEGF in patients with cancer, confirming herewith the observations that the majority of blood VEGF is contained within platelets (Benoy *et al*, 2002).

The biological significance of a higher platelet VEGF-A load in patients with cancer is not clear. Nevertheless, in patients with cancer, a worse prognosis is associated with a high platelet VEGF-A load (O'Byrne *et al*, 2000). Pinedo *et al* (1998) postulated a role of platelets in promoting angiogenesis through local release of pro-angiogenic molecules (e.g. VEGF-A, bFGF, PDGF). A net pro-angiogenic effect has recently been demonstrated, although platelets also contain anti-angiogenic proteins (TSP-1, PF-4) (Verheul *et al*, 2000b).

In our study we have shown adherence, aggregation and extra-vasation of platelets in tumours (Figure 1C). Adherence, aggregation and extra-vasation lead to activation of platelets, which is invariably associated with consequent release of pro-angiogenic molecules (e.g. VEGF-A) contained in the platelet α-granules (Verheul *et al*, 2000a). Our results may therefore partly confirm the hypothesis postulated by Pinedo and colleagues.

Interleukin-6 is also known to modulate fibrinogen expression in hepatic cells. High circulating fibrinogen levels are encountered in cancer patients (Yamaguchi *et al*, 1998) and in this study we confirmed previously found correlations between serum IL-6 and circulating plasma fibrinogen and D-dimers (Dirix *et al*, 2002). This suggests that tumour-produced IL-6 may induce fibrinogen expression in hepatic cells, herewith partly explaining the observed correlations between high ciculating IL-6 levels and circulating fibrinogen levels (*P*=0.005; *r*=0.69; Figure 3C).

Fibrinogen is under normal conditions encountered only in small quantities in the extra-cellular matrix of tumours. Tumour vessels exhibit an enhanced, VEGF-A induced, vascular permeability and kinetics of extravasation of several circulating proteins are enhanced in cancer patients, e.g. fibrinogen (Dvorak *et al*, 1984). Recent evidence, however, demonstrates that tumour cells themselves produce fibrinogen, which is consequently deposited in the extra-cellular matrix (Rybarczyk and Simpson-Haidaris, 2000).

Fibrinogen is, in the pro-coagulant environment of the tumour stroma, rapidly converted to fibrin. Interleukin-6 is able to induce tissue factor expression on e.g. monocytes and may therefore contribute to the intra-tumoural activation of coagulation (Neumann *et al*, 1997). Measuring the fibrin degradation product D-dimer in the efferent vessel draining the tumour indicates that circulating D-dimer levels are also derived from intra-tumoural fibrinogen metabolism (Figure 2D). The borderline significance found (*P*=0.08) may probably gain significant values if a higher number of patients are involved and may also partly be explained by the involvement of patients with different histology in our study. However, our results confirm previous findings of intra-tumoural activation indicated by measuring D-dimers in veins draining lung tumours (Kalweit *et al*, 2000). The higher arterial and peripheral venous D-dimer levels encountered in patients with extensive disease compared with non-metastatic patients, masking observed arterio-venous gradients compared with non-metastatic patients, suggest that fibrinogen turnover also occurs in metastatic deposits. We have detected fibrin in metastatic skin deposits from patients with breast cancer (unpublished data). The importance of plasma D-dimers is illustrated by our previous finding that high circulating D-dimer levels correlate with progression kinetics (*P*<0.0001), with tumour volume (*P*<0.0001) and with reduced overall survival in patients with metastatic breast cancer (Dirix *et al*, 2002).

In conclusion, this study provides further evidence that IL-6 may be pivotal in haemostasis and angiogenesis associated with cancer.

## References

[bib1] BanksREForbesMAKinseySEStanleyAInghamEWaltersCSelbyPJ1998 Release of the angiogenic cytokine vascular endothelial growth factor (VEGF) from platelets: significance for VEGF measurements and cancer biology Br J Cancer 77 956964 9528841 10.1038/bjc.1998.158 PMC2150108

[bib2] BenoyISalgadoRColpaertCWeytjensRVermeulenPBDirixLY2002 Serum interleukin 6, plasma VEGF, serum VEGF, and VEGF platelet load in breast cancer patients Clin Breast Cancer 2 311315 11899364 10.3816/cbc.2002.n.008

[bib3] BobikRHongYBreierGMartinJFErusalimskyJD1998 Thrombopoietin stimulates VEGF release from c-Mpl-expressing cell lines and haematopoietic progenitors FEBS Lett 423 1014 9506832 10.1016/s0014-5793(98)00056-8

[bib4] BordenECChinP1994 Interleukin-6: a cytokine with potential diagnostic and therapeutic roles J Lab Clin Med 123 824829 8201259

[bib5] CarmelietPCollenD1998 Development and disease in proteinase-deficient mice: role of the plasminogen, matrix metalloproteinase and coagulation system Thromb Res 91 255285 9772009 10.1016/s0049-3848(98)00122-4

[bib6] CarmelietPJainRK2000 Angiogenesis in cancer and other diseases Nature 407 249257 11001068 10.1038/35025220

[bib7] CohenTNahariDCeremLWNeufeldGLeviBZ1996 Interleukin 6 induces the expression of vascular endothelial growth factor J Biol Chem 271 736741 8557680 10.1074/jbc.271.2.736

[bib8] ContrinoJGoralnickSQiJHairGRicklesFRKreutzerDL1997 Fibrin induction of tissue factor expression in human vascular endothelial cells Circulation 96 605613 9244233

[bib9] De VitaFRomanoCOrdituraMGaliziaGMartinelliELietoECatalanoG2001 Interleukin-6 serum level correlates with survival in advanced gastrointestinal cancer patients but is not an independent prognostic indicator J Interferon Cytokine Res 21 4552 11177580 10.1089/107999001459150

[bib10] DirixLYSalgadoRWeytjensRColpaertCBenoyIHugetPvan DamPProveALemmensJVermeulenP2002 Plasma fibrin D-dimer levels correlate with tumour volume, progression rate and survival in patients with metastatic breast cancer Br J Cancer 86 389395 11875705 10.1038/sj.bjc.6600069 PMC2375200

[bib11] DirixLYVermeulenPBPawinskiAProveABenoyIDe PooterCMartinMVan OosteromAT1997 Elevated levels of the angiogenic cytokines basic fibroblast growth factor and vascular endothelial growth factor in sera of cancer patients Br J Cancer 76 238243 9231925 10.1038/bjc.1997.368 PMC2223937

[bib12] DvorakHFBrownLFDetmarMDvorakAM1995 Vascular permeability factor/vascular endothelial growth factor, microvascular hyperperme ability, and angiogenesis Am J Pathol 146 10291039 7538264 PMC1869291

[bib13] DvorakHFHarveyVSMcDonaghJ1984 Quantitation of fibrinogen influx and fibrin deposition and turnover in line 1 and line 10 guinea pig carcinomas Cancer Res 44 33483354 6744268

[bib14] EdwardsRLRicklesFRMoritzTEHendersonWGZacharskiLRFormanWBCornellCJForcierRJO'DonnellJFHeadleyE1987 Abnormalities of blood coagulation tests in patients with cancer Am J Clin Pathol 88 596602 3673941 10.1093/ajcp/88.5.596

[bib15] EpplerSMCombsDLHenryTDLopezJJEllisSGYiJHAnnexBHMcCluskeyERZioncheckTF2002 A target-mediated model to describe the pharmacokinetics and hemodynamic effects of recombinant human vascular endothelial growth factor in humans Clin Pharmacol Ther 71 2032 10.1067/mcp.2002.126179 12152001

[bib16] GunsiliusEPetzerAStockhammerGNussbaumerWSchumacherPClausenJGastlG2000 Thrombocytes are the major source for soluble vascular endothelial growth factor in peripheral blood Oncology 58 169174 10705245 10.1159/000012095

[bib17] HiranoT1999 Molecular basis underlying functional pleiotropy of cytokines and growth factors Biochem Biophys Res Commun 260 303308 10403765 10.1006/bbrc.1999.0609

[bib18] ImaiTKoikeKKuboTKikuchiTAmanoYTakagiMOkumuraNNakahataT1991 Interleukin-6 supports human megakaryocytic proliferation and differentiation in vitro Blood 78 19691974 1912578

[bib19] IshibashiTKimuraHShikamaYUchidaTKariyoneSHiranoTKishimotoTTakatsukiFAkiyamaY1989 Interleukin-6 is a potent thrombopoietic factor in vivo in mice Blood 74 12411244 2788464

[bib20] KalweitGAFeindtPMicekMGamsEHellsternP2000 Markers of activated hemostasis and fibrinolysis in patients with pulmonary malignancies: comparison of plasma levels in central venous and pulmonary venous blood Thromb Res 97 105111 10680641 10.1016/s0049-3848(99)00161-9

[bib21] KellarKLHooperWCBensonJM1995 MEG-01s cells have receptors for and respond to IL-3, IL-6, and SCF Exp Hematol 23 557564 7539384

[bib22] KudrykBJBiniA2000 Monoclonal antibody designated T2G1 reacts with human fibrin beta-chain but not with the corresponding chain from mouse fibrin Arterioscler Thromb Vasc Biol 20 18481849 10894828 10.1161/01.atv.20.7.1848

[bib23] LandriscinaMCassanoARattoCLongoRIppolitiMPalazzottiBCrucittiFBaroneC1998 Quantitative analysis of basic fibroblast growth factor and vascular endothelial growth factor in human colorectal cancer Br J Cancer 78 765770 9743297 10.1038/bjc.1998.575 PMC2062968

[bib24] LeungDWCachianesGKuangWJGoeddelDVFerraraN1989 Vascular endothelial growth factor is a secreted angiogenic mitogen Science 246 13061309 2479986 10.1126/science.2479986

[bib25] LjungbergBGrankvistKRasmusonT1997 Serum interleukin-6 in relation to acute-phase reactants and survival in patients with renal cell carcinoma Eur J Cancer 33 17941798 9470835 10.1016/s0959-8049(97)00179-2

[bib26] MechtcheriakovaDWlachosAHolzmullerHBinderBRHoferE1999 Vascular endothelial cell growth factor-induced tissue factor expression in endothelial cells is mediated by EGR-1 Blood 93 38113823 10339488

[bib27] MielickiWSerwaJKurzawinskiTWierzbickiR1990 Procoagulant activity of human stomach and colon cancers Oncology 47 299302 2367056 10.1159/000226837

[bib28] MohleRGreenDMooreMANachmanRLRafiiS1997 Constitutive production and thrombin-induced release of vascular endothelial growth factor by human megakaryocytes and platelets Proc Natl Acad Sci USA 94 663668 9012841 10.1073/pnas.94.2.663 PMC19570

[bib29] NagyJABrownLFSengerDRLanirNVan de WaterLDvorakAMDvorakHF1989 Pathogenesis of tumor stroma generation: a critical role for leaky blood vessels and fibrin deposition Biochim Biophys Acta 948 305326 2465781 10.1016/0304-419x(89)90004-8

[bib30] NagyJAMorganESHerzbergKTManseauEJDvorakAMDvorakHF1995 Pathogenesis of ascites tumor growth: angiogenesis, vascular remodeling, and stroma formation in the peritoneal lining Cancer Res 55 376385 7529135

[bib31] NakashimaJTachibanaMHoriguchiYOyaMOhigashiTAsakuraHMuraiM2000 Serum interleukin 6 as a prognostic factor in patients with prostate cancer Clin Cancer Res 6 27022706 10914713

[bib32] NeumannFJOttIMarxNLutherTKenngottSGawazMKotzschMSchomigA1997 Effect of human recombinant interleukin-6 and interleukin-8 on monocyte procoagulant activity Arterioscler Thromb Vasc Biol 17 33993405 9437185 10.1161/01.atv.17.12.3399

[bib33] O'ByrneKJDobbsNHarrisAL2000 Serum vascular endothelial growth factor load and interleukin-6 in cancer patients Br J Cancer 82 18951896 10839310 10.1054/bjoc.1999.1115 PMC2363222

[bib34] OguraMMorishimaYOhnoRKatoYHirabayashiNNaguraHSaitoH1985 Establishment of a novel human megakaryoblastic leukemia cell line, MEG- 01, with positive Philadelphia chromosome Blood 66 13841392 2998511

[bib35] PinedoHMVerheulHMD'AmatoRJFolkmanJ1998 Involvement of platelets in tumour angiogenesis? Lancet 352 17751777 9848370 10.1016/s0140-6736(98)05095-8

[bib36] RybarczykBJSimpson-HaidarisPJ2000 Fibrinogen assembly, secretion, and deposition into extracellular matrix by MCF-7 human breast carcinoma cells Cancer Res 60 20332039 10766195

[bib37] SalgadoRBenoyIBogersJWeytjensRVermeulenPDirixLVan MarckE2001 Platelets and vascular endothelial growth factor (VEGF): a morphological and functional study Angiogenesis 4 3743 11824377 10.1023/a:1016611230747

[bib38] SalgadoRVermeulenPBBenoyIWeytjensRHugetPVan MarckEDirixLY1999 Platelet number and interleukin-6 correlate with VEGF but not with bFGF serum levels of advanced cancer patients Br J Cancer 80 892897 10360671 10.1038/sj.bjc.6690437 PMC2362300

[bib39] SalvenPOrpanaAJoensuuH1999 Leukocytes and platelets of patients with cancer contain high levels of vascular endothelial growth factor Clin Cancer Res 5 487491 10100697

[bib40] SimpsonRJHammacherASmithDKMatthewsJMWardLD1997 Interleukin-6: structure-function relationships Protein Sci 6 929955 9144766 10.1002/pro.5560060501 PMC2143693

[bib41] SteinbergMHKeltonJGCollerBS1987 Plasma glycocalicin. An aid in the classification of thrombocytopenic disorders N Engl J Med 317 10371042 3657867 10.1056/NEJM198710223171701

[bib42] StirkCMReidAMelvinWTThompsonWD2000 Locating the active site for angiogenesis and cell proliferation due to fibrin fragment E with a phage epitope display library Gen Pharmacol 35 261267 11888682 10.1016/s0306-3623(01)00114-8

[bib43] TempferCZeislerHSliutzGHaeuslerGHanzalEKainzC1997 Serum evaluation of interleukin 6 in ovarian cancer patients Gynecol Oncol 66 2730 9234916 10.1006/gyno.1997.4726

[bib44] ThompsonWDCampbellREvansT1985 Fibrin degradation and angiogenesis: quantitative analysis of the angiogenic response in the chick chorioallantoic membrane J Pathol 145 2737 2578560 10.1002/path.1711450103

[bib45] TurleyHScottPAWattsVMBicknellRHarrisALGatterKC1998 Expression of VEGF in routinely fixed material using a new monoclonal antibody VG1 J Pathol 186 313318 10211122 10.1002/(SICI)1096-9896(1998110)186:3<313::AID-PATH188>3.0.CO;2-X

[bib46] VeikkolaTKarkkainenMClaesson-WelshLAlitaloK2000 Regulation of angiogenesis via vascular endothelial growth factor receptors Cancer Res 60 203212 10667560

[bib47] VerheulHMHoekmanKLupuFBroxtermanHJvan der ValkPKakkarAKPinedoHM2000a Platelet and coagulation activation with vascular endothelial growth factor generation in soft tissue sarcomas Clin Cancer Res 6 166171 10656446

[bib48] VerheulHMHoekmanKLuykx-de BakkerSEekmanCAFolmanCCBroxtermanHJPinedoHM1997 Platelet: transporter of vascular endothelial growth factor Clin Cancer Res 3 21872190 9815613

[bib49] VerheulHMJornaASHoekmanKBroxtermanHJGebbinkMFPinedoHM2000b Vascular endothelial growth factor-stimulated endothelial cells promote adhesion and activation of platelets Blood 96 42164221 11110694

[bib50] WertherKBülowSHesselfeldtPJespersenNSvendsenMNNielsenHJ2001 VEGF concentrations in tumour arteries and veins from patients with rectal cancer Euroconference on Angiogenesis, Parisabstract nr. C-19

[bib51] YamaguchiTYamamotoYYokotaSNakagawaMItoMOguraT1998 Involvement of interleukin-6 in the elevation of plasma fibrinogen levels in lung cancer patients Jpn J Clin Oncol 28 740744 9879291 10.1093/jjco/28.12.740

